# Supplementary material for: Coping with Space Neophobia in Drosophila melanogaster: The Asymmetric Dynamics of Crossing a Doorway to the Untrodden
Source: PLoS One. 2015 Dec 8;10(12):e0140207. doi: 10.1371/journal.pone.0140207 (PMC4672930; doi:10.1371/journal.pone.0140207)
Supplement: S1 File — Animation of the paths traced during successive entries by a specific fly in the course of a 3 days session (Video A). The paths traced by a specific fly in the vicinity of the doorway before and up until the first entry (Video B). The paths traced by a specific fly in the untrodden arena in the vicinity of the doorway before and up until the first time it resists crossing to the trodden arena (Video C). The sequence of ratios between the duration of each entry and the duration of the departure that preceded it in a selected fly (Fig A). The developmental dynamics of entry duration, departure duration and the ratio between them (Fig B). (DOCX) [file pone.0140207.s001.docx]

**Supporting information**

[Video A](https://www.youtube.com/watch?v=9nQVGxRm7Ao&feature=youtu.be).

Video A: **Animation of the paths traced during successive entries by a specific fly in the course of a 3 days session**. The current entry is colored in red. History of entries is plotted in gray. Note the progressive increase in extent and complexity of entries.

[Fly plot, going from the Small arena to the Large arena (Click to view)](http://youtu.be/rH9kOGIUx3o)

Video B related to Fig 5.**The paths traced by the same selected fly as in video A in the trodden arena in the vicinity of the doorway (in pink) until the first entry to the untrodden arena (in blue).**

[Fly plot, going from the Large arena to the Small arena (click to view)](http://youtu.be/mkkcZ9-O-QA)

Video C related to Fig 6. **All paths traced by the same selected fly as in video A upon approaching the doorway from the side of the untrodden arena**. All paths proceed to the familiar arena (in pink) until the first avoidance (in blue) of the doorway (holding back).

**
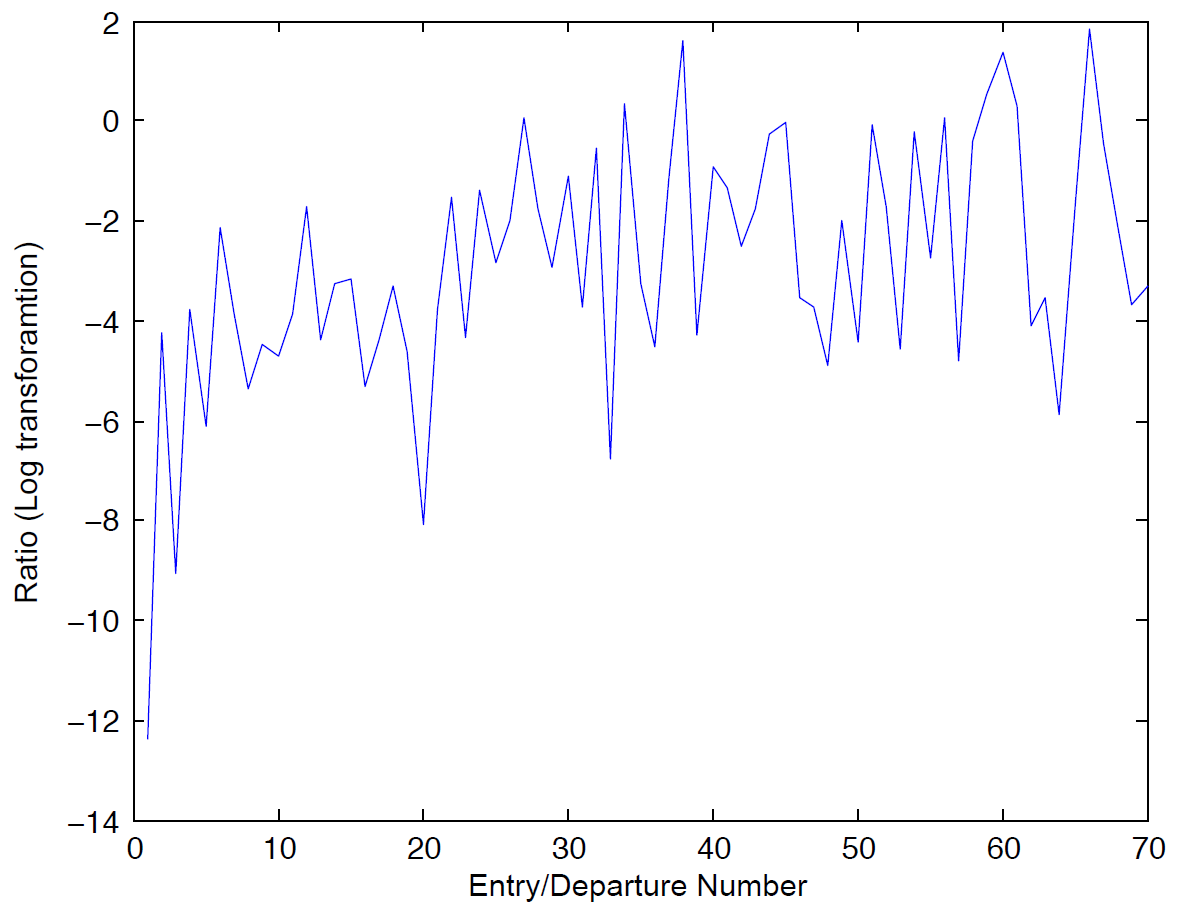
**

**Fig.A Related to fig. 7: The sequence of ratios between the duration of each entry and the duration of the departure that preceded it in a selected fly**. This plot also suggests a positive trend, implying a progressive increase in time spent in the large arena relative to time spent in the small arena. A significant positive change in the entry/departure durations ratio is evident in the fly population as a whole (p=0.0015; Fig 12). The large arena first acts as a repeller and the small arena as an attractor. The repulsive force of the large arena and the attractive force of the small arena are gradually diminished reducing the asymmetry in the behavior in the two compartments.


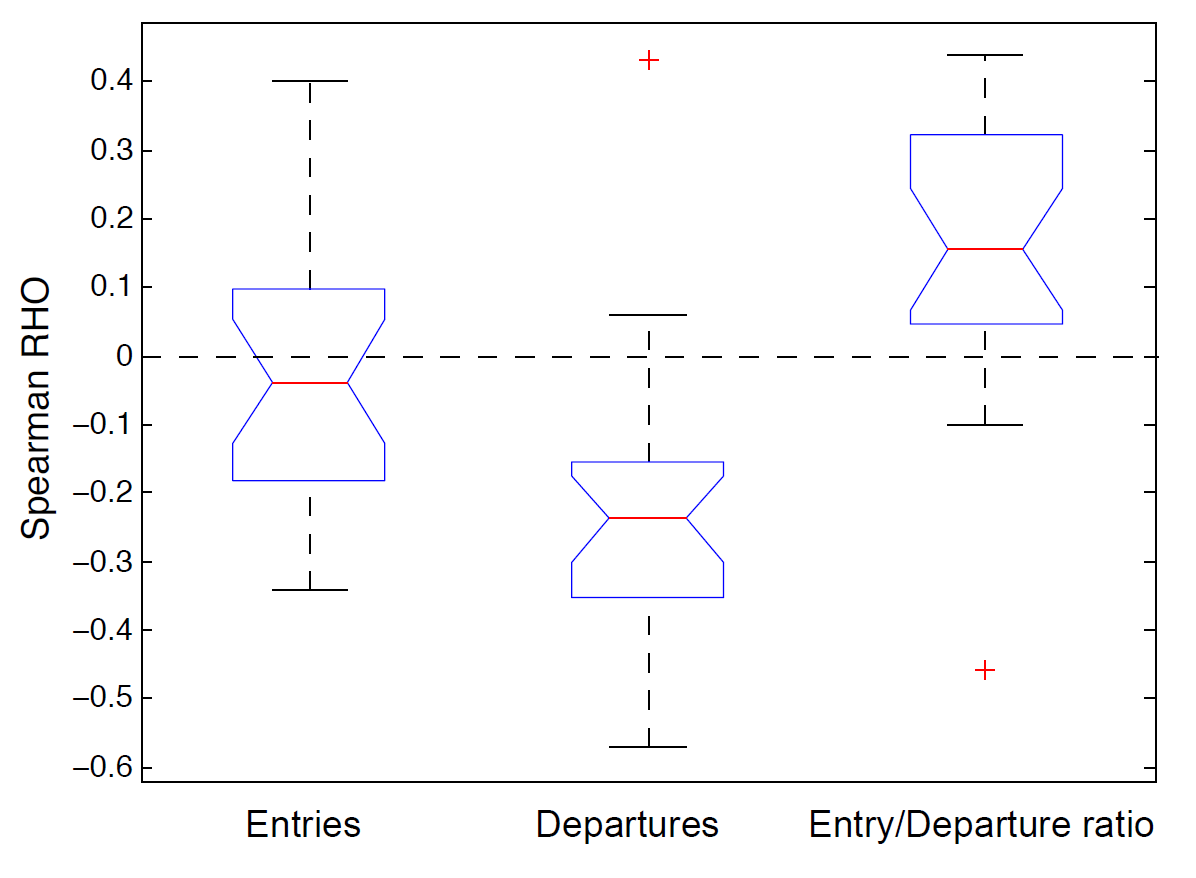


**Fig.B related to Fig.8: The developmental dynamics of entry duration, departure duration and the ratio between them.** As shown by the Spearman RHO, entry duration does not seem to change across the sequence of entries (p=1) while departure duration decreases across the sequence of departures (p=0.00028), resulting in an increase across the sequence of entry/departure duration ratios (p=0.0015).
